# ECG-based detection of occlusion myocardial infarction: a dedicated deep neural network versus multimodal large language models and physicians — a retrospective diagnostic accuracy study

**DOI:** 10.1186/s12873-026-01716-3

**Published:** 2026-08-07

**Authors:** Emin Hüseyin Akar, Kamil Kokulu, Ekrem Taha Sert

**Affiliations:** 1 Department of Emergency Medicine, Aksaray Training and Research Hospital, Aksaray, 68100 Turkey; 2 Department of Emergency Medicine, Aksaray University School of Medicine, Aksaray, Turkey

**Keywords:** Artificial intelligence, Large language models, Electrocardiography, Occlusion myocardial infarction, ST elevation myocardial infarction, Clinical decision support systems

## Abstract

**Background:**

Accurate electrocardiogram (ECG) interpretation for acute coronary occlusion is a critical, time-sensitive task in emergency care. The field is increasingly shifting from the ST-segment elevation myocardial infarction paradigm to the broader concept of occlusion myocardial infarction (OMI). This study compared the diagnostic accuracy of a dedicated occlusion-detection deep neural network, Queen of Hearts (QoH), two general-purpose multimodal large language models (LLMs), and emergency physicians for ECG-based OMI detection. Response consistency and confidence calibration of the LLMs were also evaluated.

**Methods:**

This retrospective diagnostic accuracy study used 36 twelve-lead ECGs from patients referred for emergent coronary angiography, including 24 with angiographically confirmed acute coronary occlusion and 12 without. Five emergency medicine specialists, five emergency medicine residents, and two LLMs, ChatGPT 5.2 and Gemini 3 Pro, interpreted all ECGs under a standardized moderate-risk acute coronary syndrome scenario. The same ECGs were submitted to QoH. Each LLM evaluated the set five times in separate sessions, whereas QoH was queried once because it provides deterministic output. In the primary head-to-head analysis, each interpreter or interpreter group contributed a single decision per ECG. Areas under the curve (AUCs) were compared using the DeLong test, binary metrics using the exact McNemar test, and reliability using Fleiss’ kappa.

**Results:**

QoH showed the highest discrimination for OMI (AUC 0.96), followed by ChatGPT (0.81) and Gemini (0.62). QoH also achieved the highest sensitivity (95.8%, missing one of 24 occlusions) and accuracy (86.1%), with significantly higher sensitivity than emergency specialists (*p* = 0.016). Among physicians, specialists performed best (accuracy 75.0%; sensitivity 66.7%; specificity 91.7%). ChatGPT showed high specificity (91.7%) but low sensitivity (54.2%), whereas Gemini performed least well overall (accuracy 55.6%). The LLMs produced variable interpretations across repeated queries (Fleiss’ kappa 0.24–0.49), and Gemini showed marked overconfidence (Brier score 0.40).

**Conclusions:**

In this OMI-focused study, QoH showed the highest discrimination and sensitivity for acute coronary occlusion. General-purpose LLMs showed clinically important limitations and do not currently support autonomous OMI diagnosis. These findings support further evaluation of task-specific models for ECG-based occlusion detection and suggest, at most, an adjunctive, human-supervised role for current general-purpose LLMs.

**Supplementary Information:**

The online version contains supplementary material available at https://doi.org/10.1186/s12873-026-01716-3.

## Introduction

ST-segment elevation myocardial infarction (STEMI) is a “time-critical” medical emergency where the time from diagnosis to reperfusion significantly impacts clinical outcomes [1]. Therefore, current guidelines recommend the system-level optimization of early electrocardiogram (ECG) diagnosis and rapid reperfusion strategies for patients with suspected STEMI [1, 2]. The first step in both prehospital and hospital-based STEMI care processes is to obtain an ECG and establish a STEMI diagnosis within 10 min of the first medical contact [1].

However, diagnosing STEMI is challenging, especially in emergency department (ED) and prehospital scenarios where decisions must be made relying solely on the ECG with limited clinical information [3, 4]. Part of this diagnostic difficulty arises from STEMI mimickers, a concept emphasized in appropriate catheterization laboratory activation frameworks [5]. Conditions such as early repolarization, left ventricular hypertrophy, left bundle branch block, and pericarditis can cause ST elevation on the ECG in the absence of acute coronary occlusion, leading to “false-positive” STEMI evaluations and unnecessary invasive procedures [6]. A more critical diagnostic problem is missing the diagnosis on the initial ECG. In a national cohort of patients discharged with a final diagnosis of myocardial infarction, 29.9% had an initial hospital diagnosis that differed from their final diagnosis, and 3.3% of those ultimately diagnosed with STEMI had been admitted under an initial diagnosis other than myocardial infarction [7]. Furthermore, another study reported the sensitivity of physicians in recognizing “true STEMI” ECGs as 65% [8]. Additionally, inter-reader agreement among physicians in interpreting ECGs with suspected STEMI has been found to be low [8]. For all these reasons, providing clinical decision support to frontline physicians is critical to reducing misdiagnoses and improving STEMI management. Notably, the ST-segment elevation paradigm itself has limitations: millimeter-based ST-elevation criteria identify only approximately 43% of acute coronary occlusions, and the field is increasingly adopting the occlusion/non-occlusion myocardial infarction (OMI/NOMI) framework, in which OMI encompasses both classic STEMI and STEMI-equivalent patterns [5, 9, 10].

This area of need has increased interest in decision-support technologies for ECG interpretation. Although traditional computerized ECG interpretation systems have been in use for a long time, it has been emphasized that they require physician supervision in clinical practice due to diagnostic limitations [11, 12]. In recent years, multimodal large language models (LLMs) have rapidly become widespread in clinical applications owing to their ability to process visual inputs, and research in this area has increased significantly [13–16]. Previous studies have reported that LLMs like ChatGPT perform comparably to physicians in answering multiple-choice ECG questions [14, 17]. Moreover, a study including patients presenting to the ED reported that ChatGPT demonstrated moderate accuracy in ECG interpretation [13]. However, the performance and reliability of widely used LLMs such as ChatGPT and Gemini in high-risk visual pattern recognition tasks, such as diagnosing STEMI, remain largely untested. In contrast, dedicated deep neural networks trained specifically to detect acute coronary occlusion have achieved high diagnostic accuracy, yet image-based general-purpose LLMs have not been benchmarked against such purpose-built models for this task [18].

The aim of this study was to compare the diagnostic accuracy of a dedicated occlusion-detection deep neural network (Queen of Hearts), two general-purpose multimodal LLMs (ChatGPT 5.2 and Gemini 3 Pro), and emergency physicians for the detection of OMI from isolated ECGs. The secondary objective was to measure the response consistency (intra-rater reliability) of the large language models.

## Methods

### Study Design

This study is a retrospective, observational diagnostic accuracy study designed to compare the accuracy of artificial intelligence-based LLMs (ChatGPT 5.2 and Gemini 3 Pro) and emergency physicians in diagnosing OMI. The study utilized 36 12-lead ECGs previously compiled by McCabe et al. to evaluate the accuracy of physicians in interpreting STEMI ECGs, which were made publicly available online as supplementary material [8]. The ECGs were obtained from real patients referred for emergent coronary angiography. The source dataset used historical STEMI terminology; however, angiography-positive cases included ECGs consistent with both classic STEMI and STEMI-equivalent occlusion patterns. Therefore, the target condition in the present analysis was defined as angiographically confirmed OMI/acute coronary occlusion. The target condition was acute coronary occlusion, i.e., OMI, defined as an angiographically confirmed acute culprit occlusion (or less than TIMI grade III coronary flow); this construct corresponds to the contemporary OMI/NOMI paradigm and served as the reference standard for all interpreters. According to the emergent coronary angiography (reference standard) results, 24 (67%) of the 36 ECGs were considered consistent with OMI, while in 12 (33%), no culprit lesion was detected, thereby ruling out OMI. The study protocol was approved by the institutional review board (approval no: 2026/42).

### Physician Interpreters and Data Collection

The physician interpreter group consisted of five Emergency Medicine (EM) Specialists and five EM Residents actively working in clinical practice. The methodology of McCabe et al. was modified and utilized in this study [8]. To measure the physicians’ ECG interpretation performances entirely independently of clinical variables such as the patient’s risk stratification or pretest probability, a standard clinical scenario was used for all cases. All cases were described as a “patient with a moderate risk of acute coronary syndrome,” and participants were not provided with any additional clinical details such as age, sex, or symptoms. Additionally, the interpreters were blinded (not informed) to the fact that these cases were selected from patients who had an indication for emergent angiography. The ECGs were presented to the physicians in an online survey format via Google Forms (Alphabet Inc., California, USA) between February 5 and 6, 2026. Adhering to the original study, the introductory instructions of the survey included the following statement: “We recognize that the activation of the catheterization laboratory is a multifactorial clinical decision; however, our expectation from you in this study is to evaluate whether the ECG you see meets the criteria for an acute coronary occlusion (STEMI).” Following each ECG image, the physicians were asked to answer a single question: “Based on the ECG above, is there an acute coronary occlusion causing a STEMI?“. This wording was retained verbatim from the original McCabe instrument to preserve comparability; however, the target condition in the present analysis was angiographically defined OMI/acute coronary occlusion. The response options were strictly “Yes” and “No”. Each physician interpreted the set of 36 ECGs only once.

### AI Models and Prompting Strategy

Two different LLMs were evaluated in the study: ChatGPT 5.2 (OpenAI, San Francisco, USA) and Gemini 3 Pro (Google, California, USA). The ECG analyses by the AI models were conducted simultaneously with the human physicians on February 5–6, 2026.

To compare the performance of the AI models with human physicians on a fair and standardized platform, all cases were presented to the models using the same “patient with a moderate risk of acute coronary syndrome” clinical scenario. The aim was for the models, like the physicians, to focus solely on analyzing electrocardiographic patterns (pattern recognition), devoid of demographic or additional clinical data. To minimize the risk of hallucination and ensure they reflected the clinical perspective of an “Expert Cardiologist,” a structured, single-step prompt was designed. The prompt informed the models that the study was retrospective and involved no real-time patient risk. Using the same wording as the physician survey, the models were asked whether the ECG met criteria for an acute coronary occlusion. The models were required to generate a standard output format containing the following headings: 1) A short technical summary of ECG findings (Rhythm, ST segments, T waves, and reciprocal changes), 2) A definitive binary diagnosis (strictly in ‘YES’ or ‘NO’ format), 3) A confidence score for the diagnosis (ranging from 0 to 100%). The full prompt text used is provided as Supplementary Material-1.

To objectively measure the response stability and reproducibility of the LLMs, each ECG was queried to the models five times in different chat sessions [14, 17, 19–21]. In each new evaluation, a separate chat session was opened to prevent the models from remembering their previous interpretations and to ensure the responses did not influence one another.

In addition to the two general-purpose LLMs, the same 36 ECG images were submitted to Queen of Hearts (QoH; PMcardio, Powerful Medical), an image-based deep neural network specifically trained to detect acute coronary occlusion. Each ECG image was submitted once because QoH provides a deterministic output for a given input; therefore, repeated submissions were not part of the primary analysis. The application-provided binary classification for acute coronary occlusion and the reported confidence/probability score were recorded. For the receiver operating characteristic analysis, a continuous occlusion score was derived from this reported confidence, analogous to the procedure used for the large language models (described in the Statistical Analysis section).

### Statistical analysis

Data were analyzed using Python 3.8 (Python Software Foundation) and relevant libraries (SciPy, Statsmodels, Scikit-learn). With coronary angiography results considered the “reference standard,” the following diagnostic performance metrics (Accuracy, Sensitivity, Specificity, positive predictive value, negative predictive value, positive likelihood ratio, negative likelihood ratio) were calculated for each physician interpreter and AI model, and presented with 95% Confidence Intervals (CIs). The diagnostic odds ratio (DOR) was also calculated as a summary measure of overall diagnostic utility. The primary analysis reduced each interpreter to a single decision per ECG (the majority vote across the five runs or readers for the LLMs and physicians, and the single deterministic output for QoH), so that the 36 ECGs were treated as independent units; AUCs were compared with the DeLong test, sensitivity, specificity and accuracy with the exact McNemar test, and proportions with Clopper–Pearson 95% confidence intervals. The continuous occlusion score used for the ROC analysis was obtained, for the LLMs, as the mean of the five per-run signed confidence values and, for QoH, from its reported confidence. For each LLM run, the signed confidence value was defined as the reported confidence when the model answered ‘Yes’ and as 1 minus the reported confidence when it answered ‘No’. Analyses pooling all repeated interpretations are reported in the Supplement, where 95% confidence intervals were obtained by a cluster bootstrap (10,000 resamples, with the ECG as the resampling unit) and between-group differences by generalized estimating equations with cluster-robust standard errors clustered on ECG. The discriminative performances of the models for OMI were visualized using Receiver Operating Characteristic (ROC) curves, and Area Under the Curve (AUC) values were calculated. Inter-rater reliability and the consistency among the consecutive evaluations of the AI models were analyzed using Fleiss’ Kappa statistics. To quantitatively evaluate the calibration of the probabilistic predictions and the overconfidence in the certainty levels of the artificial intelligence models, the Brier Score was calculated. The Brier score is a metric that ranges from 0 to 1, where a value of 0 represents flawless prediction calibration (perfect confidence), and a value of 1 represents the worst possible calibration. In a binary (yes/no) diagnostic test such as the one in our study, the theoretical Brier score for an evaluator making completely random guesses is considered to be 0.25. The Mann-Whitney U test was used to compare the confidence scores of the AI models. A p-value of < 0.05 was considered statistically significant in all tests.

## Results

### Overall Diagnostic Performance

A total of 36 ECGs were included in the study. Each ECG was interpreted once by QoH, five times in separate sessions by ChatGPT 5.2 and Gemini 3 Pro, and once by each of five EM specialists and five EM residents. For the primary head-to-head analysis, each interpreter was reduced to a single decision per ECG (Methods); the resulting confusion-matrix components are summarized in Table 1, and the corresponding diagnostic performance metrics, including discrimination (AUC), are detailed in Table 2. Analyses pooling all repeated interpretations are provided in Supplementary Tables 1 and 2.

**Table 1:** Confusion-matrix components for case-level detection of occlusion myocardial infarction

ECG Interpreter	Number of ECG Interpretations	True Positives	False Negatives	False Positives	True Negatives
Queen of Hearts	36	**23**	**1**	**4**	**8**
ChatGPT 5.2	36	13	11	1	11
Gemini 3 Pro	36	14	10	6	6
Emergency Medicine Specialists	36	16	8	1	11
Emergency Medicine Residents	36	15	9	6	6

False Positives (Overcall): Over-diagnosis, False Negatives (Undercall): Missed diagnosis. One decision per ECG on the common 36 ECGs (24 OMI, 12 non-OMI). LLMs/physicians: majority vote across 5 runs/readers; Queen of Hearts: single deterministic output. OMI = occlusion myocardial infarction (angiographically confirmed acute culprit occlusion)

**Table 2:** Primary case-level diagnostic performance for detection of occlusion myocardial infarction

ECG Interpreter	AUC (95% CI)	Accuracy (95% CI)	Sensitivity (95% CI)	Specificity (95% CI)	+LR (95% CI)	–LR (95% CI)	DOR (95% CI)
Queen of Hearts (DNN)	0.962 (0.908–1.000)	86.1% (70.5–95.3)	95.8% (78.9–99.9)	66.7% (34.9–90.1)	2.88 (1.29–6.43)	0.06 (0.01–0.44)	46.0 (4.5–474.9)
ChatGPT 5.2	0.812 (0.656–0.969)	66.7% (49.0–81.4)	54.2% (32.8–74.4)	91.7% (61.5–99.8)	6.50 (0.96–44.00)	0.50 (0.31–0.80)	13.0 (1.4–117.2)
Gemini 3 Pro	0.615 (0.401–0.829)	55.6% (38.1–72.1)	58.3% (36.6–77.9)	50.0% (21.1–78.9)	1.17 (0.60–2.26)	0.83 (0.40–1.74)	1.4 (0.35–5.63)
Emergency Specialists	— (operating point)	75.0% (57.8–87.9)	66.7% (44.7–84.4)	91.7% (61.5–99.8)	8.00 (1.20–53.37)	0.36 (0.20–0.66)	22.0 (2.4–201.8)
Emergency Residents	— (operating point)	58.3% (40.8–74.5)	62.5% (40.6–81.2)	50.0% (21.1–78.9)	1.25 (0.66–2.38)	0.75 (0.35–1.61)	1.7 (0.4–6.8)

Primary analysis. AUCs were calculated from continuous scores for the AI models: the application-reported confidence/probability score for QoH and the mean signed confidence across five runs for the LLMs. Binary diagnostic metrics were calculated at the operating point: the application-provided binary classification for QoH, and majority-vote decisions for the LLMs and physician groups. Confidence intervals were calculated using the DeLong method for AUCs, the Clopper–Pearson method for proportions, and the log method for likelihood ratios and diagnostic odds ratios. Diagnostic odds ratio, calculated as + LR/−LR and equivalently as (true positives × true negatives)/(false positives × false negatives). AUC, area under the curve; CI, confidence interval; DNN, deep neural network; DOR, diagnostic odds ratio; LLM, large language model; QoH, Queen of Hearts; +LR/−LR, positive/negative likelihood ratio. Pairwise comparisons are presented in Table 3

**Fig. 1:**
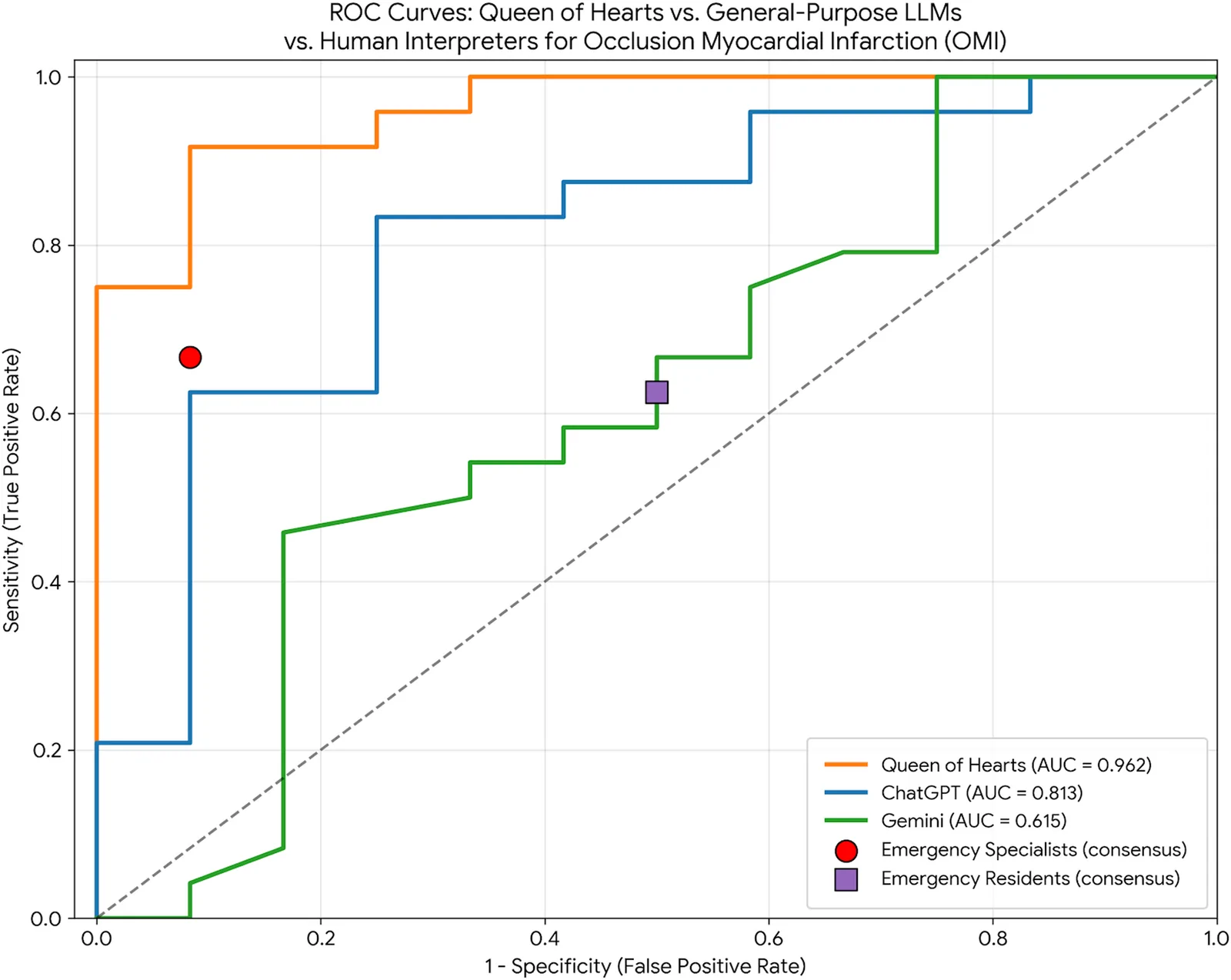
Receiver Operating Characteristic (ROC) curves for the detection of occlusion myocardial infarction (OMI) on the common set of 36 ECGs. OMI was defined as angiographically confirmed acute culprit occlusion or less than TIMI grade III flow. The orange, blue, and green curves show the discrimination of Queen of Hearts (AUC = 0.96), ChatGPT (AUC = 0.81), and Gemini (AUC = 0.62), respectively. The red circle and the purple square mark the single operating points (majority-vote consensus) of the emergency medicine specialists and residents; because these groups provide a binary decision, they are represented by a single point rather than a curve. The dashed gray diagonal represents the reference line for random guessing (AUC = 0.50)

On the common set of 36 ECGs, QoH showed the highest discrimination for OMI (AUC 0.96; 95% CI: 0.91–1.00), followed by ChatGPT (AUC 0.81; 95% CI: 0.66–0.97) and Gemini (AUC 0.62; 95% CI: 0.40–0.83) (Fig. 1). QoH also achieved the highest accuracy (86.1%) and the highest sensitivity for OMI (95.8%), missing only one of the 24 occlusions, at a specificity of 66.7%. Among the human interpreters, EM specialists performed best (accuracy 75.0%; sensitivity 66.7%; specificity 91.7%), ahead of EM residents (accuracy 58.3%). The two LLMs differed in profile: ChatGPT combined high specificity with low sensitivity (91.7% and 54.2%, respectively), whereas Gemini performed least well overall (accuracy 55.6%; sensitivity 58.3%; specificity 50.0%). Full operating characteristics for all interpreters are presented in Table 2.

### Pairwise Comparisons Between Groups

Pairwise comparisons on the 36-ECG set are presented in Table 3. The most consistent finding was the superior sensitivity of QoH: its sensitivity for OMI was significantly higher than that of every other interpreter — ChatGPT (*p* = 0.006), Gemini (*p* = 0.004), EM specialists (*p* = 0.016), and EM residents (*p* = 0.008). QoH also showed significantly higher discrimination than Gemini (ΔAUC = 0.35; DeLong *p* = 0.001) and significantly higher accuracy than Gemini (*p* = 0.003) and residents (*p* = 0.006).

**Table 3:** Pairwise comparisons of case-level diagnostic performance between interpreters

Comparison	ΔAUC (DeLong *p*)	Accuracy (McNemar *p*)	Sensitivity (McNemar *p*)	Specificity (McNemar *p*)
Queen of Hearts vs. ChatGPT	+ 0.149 (0.087)	0.144	0.006*	0.375
Queen of Hearts vs. Gemini	+ 0.347 (0.001*)	0.003*	0.004*	0.625
Queen of Hearts vs. Specialists	— (operating pt)	0.344	0.016*	0.250
Queen of Hearts vs. Residents	— (operating pt)	0.006*	0.008*	0.625
ChatGPT vs. Gemini	+ 0.198 (0.064)	0.455	1.000	0.063

Head-to-head pairwise tests on the common 36 ECGs. DeLong for paired ROC AUC; exact McNemar for paired binary decisions (sensitivity on 24 positives, specificity on 12 negatives). * *p* < 0.05. AUC, area under the curve

By contrast, the differences between QoH and ChatGPT, and between QoH and EM specialists, did not reach statistical significance for AUC or accuracy (for example, QoH versus ChatGPT, ΔAUC = 0.15, *p* = 0.087; accuracy *p* = 0.144), reflecting the limited power of a 36-ECG sample for these closer comparisons; the specificity comparisons, based on only 12 non-OMI ECGs, were likewise non-significant.

The two LLMs did not differ significantly in sensitivity (*p* = 1.000) or overall accuracy (*p* = 0.455); ChatGPT showed numerically higher specificity than Gemini (91.7% vs. 50.0%), a difference that approached but did not reach significance in this small sample (*p* = 0.063). The full set of pooled, repeated-interpretation comparisons — including the significant superiority of EM specialists over Gemini and over residents — is provided in Supplementary Table 2.

### AI Confidence Analysis

When analyzing the confidence scores of the artificial intelligence models in their own decisions, differences in calibration were observed. ChatGPT’s confidence was statistically lower on incorrect than on correct interpretations (Mean: 72.7%, Median: 71.0% vs. Mean: 75.1%, Median: 75.0%; *p* = 0.002); although statistically detectable, this difference of approximately 2.4% points was modest in magnitude.

In contrast, Gemini showed a pattern of clinically concerning overconfidence. The model stated that it was almost completely certain of its decisions both when providing a correct diagnosis (Mean: 93.9%, Median: 95.0%) and when providing an incorrect diagnosis (Mean: 92.7%, Median: 95.0%). Although there is no meaningful difference between these two scenarios in terms of clinical practice, a statistical difference was still detected (*p* = 0.038).

When the algorithmic probability calibrations of the models were analyzed using the Brier Score (a lower score indicates better calibration), ChatGPT (0.230) showed significantly better calibration than Gemini (0.400) (*p* < 0.001). Notably, Gemini’s Brier score exceeded the value of 0.25 expected from random guessing, indicating frank miscalibration, whereas ChatGPT’s score was only marginally below this threshold.

### Inter-rater Reliability

Fleiss’ kappa coefficients calculated to assess the consistency of interpretations of the same ECGs across repeated runs or individual readers are presented in Table 4. The highest agreement was observed across the repeated assessments of ChatGPT, with a Fleiss’ κ of 0.494 (95% CI: 0.313–0.655), indicating moderate agreement. Agreement among EM specialists was fair, with a Fleiss’ κ of 0.399 (95% CI: 0.226–0.555), as was agreement across Gemini assessments, with a Fleiss’ κ of 0.244 (95% CI: 0.089–0.389). Agreement among EM residents was slight, with a Fleiss’ κ of 0.086 (95% CI: −0.044 to 0.211), while agreement across all human interpreters was also slight, with a Fleiss’ κ of 0.119 (95% CI: 0.024–0.214). Because QoH provides a single deterministic output for a given input, repeated-run reliability was not assessed, and Fleiss’ κ was not applicable.

**Table 4:** Response consistency and inter-rater reliability for ECG interpretation

ECG Interpreter	No. of raters / runs	Fleiss’ κ (95% CI)	Agreement Level
Queen of Hearts (DNN)	1	Not applicable (single deterministic run)	Not applicable; single deterministic output.
ChatGPT 5.2	5	0.494 (0.313–0.655)	Moderate
Emergency Specialists	5	0.399 (0.226–0.555)	Fair
Gemini 3 Pro	5	0.244 (0.089–0.389)	Fair
Emergency Residents	5	0.086 (-0.044–0.211)	Slight
All human interpreters	10	0.119 (0.024–0.214)	Slight

Fleiss’ κ for agreement across repeated runs (LLMs) or readers (physicians) per ECG; 95% CI by bootstrap over ECGs (5,000 resamples). κ, kappa

## Discussion

In this study, we compared a dedicated occlusion-detection deep neural network (QoH), two general-purpose multimodal LLMs (ChatGPT 5.2 and Gemini 3 Pro), and emergency physicians for the detection of OMI from isolated ECGs. Our principal finding is that the dedicated model showed the strongest overall diagnostic profile, particularly for sensitivity: QoH achieved the highest discrimination (AUC 0.96) and the highest sensitivity for OMI (95.8%), missing only one of the 24 occlusions. Its sensitivity was significantly higher than that of every other interpreter, including EM specialists — a clinically critical advantage, since a missed occlusion is the most dangerous error in this setting. Among the general-purpose models, the two LLMs showed contrasting but clinically limited profiles: ChatGPT combined relatively high specificity (91.7%) with low sensitivity (54.2%), whereas Gemini performed least well overall. These results indicate that, for visual OMI recognition, current general-purpose LLMs remain clinically limited, particularly because of low sensitivity and variable reproducibility, whereas the dedicated model showed the most favorable sensitivity profile.

In the literature, it has been reported that the diagnostic accuracy of clinicians in identifying STEMI from isolated ECGs ranges between 69% and 71% [8, 22]. However, when angiographically defined OMI is the reference construct, the sensitivity of conventional STEMI-based interpretation is substantially lower: among the 136 of 2486 patients (5.5%) with a totally occluded culprit lesion in the prospective study by Hillinger et al. [23], blinded cardiologist visual ECG interpretation identified only 49% as STEMI in the occlusion-framed reanalysis by Meyers and Smith [24], underscoring the persistent STEMI–OMI gap. The performance of the physicians in our study (EM specialists 75.0%, residents 58.3%) is broadly consistent with these data. When examining the ECG interpretation performances of LLMs in the current literature, our findings show a strict alignment with recent studies. For instance, in a study by Günay et al. comparing GPT-4, GPT-4o, and Gemini models with EM specialists and cardiologists, it was found that cardiologists and EM specialists outperformed all general AI models on routine ECGs [17]. Similarly, in a recent study investigating the success of multimodal LLMs in diagnosing myocardial infarction from ECGs, the diagnostic accuracy of ChatGPT was reported as 65.95%, and its specificity as 76.2% [25]. In a study conducted in the ED by Zaboli et al., it was reported that ChatGPT’s sensitivity in interpreting the ST segment on ECGs was low, but its specificity was quite high [13]. ChatGPT’s pooled accuracy and specificity in our study (Supplementary Table 1) are in close agreement with these data.

Our findings illustrate a consistent gap between general-purpose multimodal models and purpose-built, narrow occlusion-detection networks. QoH reached an AUC of 0.96 for OMI, whereas the general-purpose LLMs reached 0.81 (ChatGPT) and 0.62 (Gemini); this mirrors prior reports in which occlusion-trained networks achieved an AUC above 0.90 for angiographically confirmed occlusion [18]. A further distinction concerns output stability: whereas the LLMs produced variable interpretations across repeated queries of identical ECGs (Fleiss’ κ 0.24–0.49), QoH provides a deterministic output for a given input; therefore, repeated submissions were not part of the primary analysis. Two recent studies provide relevant context for these observations: physicians and the same occlusion model were compared on STEMI-equivalent and mimic ECGs [26], and general-purpose LLMs and the occlusion model were compared after cardiac arrest against an angiographic outcome [27]. Together, these data suggest that, for the specific task of OMI detection from the ECG, task-specific models outperform current general-purpose LLMs and show significantly higher sensitivity than human interpreters, although not all accuracy and AUC comparisons reached statistical significance.

In the context of artificial intelligence, one of the most striking findings of our study is the profound difference in the “confidence” levels the models have in their own decisions and the issue of calibration. In medical applications, a major concern with LLMs is that they may generate incorrect or unsupported explanations while presenting them with high confidence. Indeed, in the study by Lee et al., when the reasoning behind the LLMs’ ECG diagnoses was analyzed, it was found that Gemini produced completely inaccurate arguments in 54% of cases, and ChatGPT in 58% [25]. In our study, Gemini showed a similar pattern of clinically concerning overconfidence and poor calibration by reporting a median confidence score of 95.0% even in cases where it provided an incorrect diagnosis. In contrast, ChatGPT showed somewhat better calibration, with a small but statistically significant reduction in its confidence score in erroneous diagnoses. As pointed out by Zhu et al., deploying multimodal models autonomously in EDs poses serious risks unless their quantitative measurement (e.g., millimetric ST elevation counting) and calibration capabilities are improved [28].

Considering the clinical implications, the dedicated occlusion model (QoH), with its very high sensitivity for OMI and correspondingly low negative likelihood ratio, showed a profile consistent with a potential rule-out aid for acute coronary occlusion, although this requires prospective validation. By contrast, current general-purpose LLMs do not yet appear safe as autonomous decision-makers for OMI diagnosis. ChatGPT’s comparatively high specificity and lower false-positive rate relative to residents suggest a possible supporting role as a “second reader” to help prevent unnecessary catheterization laboratory activations, but only as an adjunct; Gemini’s overconfidence and poor calibration further argue against autonomous clinical use. Realizing any such role will require prospective validation and improved calibration.

### Limitations

Our study has certain limitations. First, the study sample (36 ECGs) is relatively small; although it enabled a direct comparison with a published physician benchmark on identical ECGs, it is underpowered for the closer pairwise contrasts — for example, the large numerical AUC advantage of QoH over ChatGPT did not reach statistical significance. Moreover, because all cases came from patients referred for emergent angiography, the non-OMI ECGs were enriched for STEMI mimics, which increases case difficulty and limits the generalizability of the specificity estimates to unselected emergency populations. Second, the ECGs were presented with a standard clinical scenario (“moderate risk patient”) and did not reflect real-time patient-physician interaction or dynamic ECG changes. This lack of dynamic clinical context may have negatively affected the sensitivity of the LLMs. Third, since artificial intelligence systems are continuously updated, the performance of the versions used in this study (ChatGPT 5.2 and Gemini 3 Pro) may vary in the future. Finally, because each ECG was evaluated repeatedly, the primary analysis used a single decision per ECG to keep the cases independent, with the pooled analyses reported using cluster-aware inference in the Supplement; in addition, the QoH continuous score was derived from the application’s reported confidence rather than the raw model output.

## Conclusions

In conclusion, on a set of 36 angiographically adjudicated ECGs, QoH showed the most favorable diagnostic profile for OMI detection, particularly in terms of sensitivity, which exceeded that of every comparator, including emergency specialists. The general-purpose LLMs performed less well and showed contrasting limitations — ChatGPT favouring specificity over sensitivity, and Gemini combining the weakest overall accuracy with marked overconfidence — and are therefore not suitable for autonomous OMI diagnosis. These findings support a role for task-specific models and, at most, an adjunct (‘human-in-the-loop’ or ‘second-reader’) role for current general-purpose LLMs, pending prospective validation.

## Supplementary Information

Below is the link to the electronic supplementary material.

Supplementary Material 1

Supplementary Material 2

Supplementary Material 3

## Data Availability

The datasets used and/or analysed during the current study are available from the corresponding author on reasonable request.

## Abbreviations

AI: Artificial intelligence
AUC: Area Under the Curve
CIs: Confidence Intervals
ECG: Electrocardiogram
ED: Emergency department
EM: Emergency Medicine
LLMs: Large language models
LR−: Negative likelihood ratio
LR+: Positive likelihood ratio
NOMI: Non-occlusion myocardial infarction
NPV: Negative predictive value
OMI: Occlusion myocardial infarction
PPV: Positive predictive value
QoH: Queen of Hearts
ROC: Receiver Operating Characteristic
STEMI: ST-segment elevation myocardial infarction
