# Supplementary material for: ECG-based detection of occlusion myocardial infarction: a dedicated deep neural network versus multimodal large language models and physicians — a retrospective diagnostic accuracy study
Source: BMC Emerg Med. 2026 Aug 7;26:237. doi: 10.1186/s12873-026-01716-3 (PMC13545760; doi:10.1186/s12873-026-01716-3)
Supplement: Supplementary file 1 [file 12873_2026_1716_MOESM1_ESM.docx]

**The full prompt text:**

CONTEXT: This is a retrospective scientific study using anonymized historical data. There is NO real patient currently at risk.

ROLE: Act as an expert Cardiologist.

STANDARD CLINICAL CONTEXT:

Treat this case as a "patient with moderate risk of acute coronary syndrome." This context is provided to isolate ECG interpretation characteristics.

STUDY INSTRUCTIONS:

"We recognize that activation of the cardiac catheterization team is a clinical decision; please focus on whether or not the ECG in question meets your diagnostic criteria for an acutely blocked coronary artery (a STEMI)."

TASK:

You will perform a two-step evaluation based on the instructions above.

STEP 1: VISUAL ANALYSIS

First, analyze the ECG findings (Rhythm, ST-segments, T-waves, reciprocal changes) strictly.

STEP 2: FINAL DECISION

Answer the specific study question below based on your analysis and the "moderate risk" context.

QUESTION: "Based on the ECG above, is there a blocked coronary artery present causing a STEMI? (please provide your best guess)."

OUTPUT FORMAT:

Please present your final response strictly in the following format:

ECG FINDINGS: [Short technical summary of findings]

DIAGNOSIS: [Write only 'YES' or 'NO']

CONFIDENCE: [0-100%]
