# Supplementary material for: ECG-based detection of occlusion myocardial infarction: a dedicated deep neural network versus multimodal large language models and physicians — a retrospective diagnostic accuracy study
Source: BMC Emerg Med. 2026 Aug 7;26:237. doi: 10.1186/s12873-026-01716-3 (PMC13545760; doi:10.1186/s12873-026-01716-3)
Supplement: Supplementary file 2 [file 12873_2026_1716_MOESM2_ESM.docx]

**Supplementary Table 1.** Pooled repeated-interpretation diagnostic performance for detection of occlusion myocardial infarction

| **ECG Interpreter** | **N** | **Accuracy** | **Sensitivity** | **Specificity** | **+LR** | **–LR** | **PPV** | **NPV** |  |
| --- | --- | --- | --- | --- | --- | --- | --- | --- | --- |
| **A. Pooled across all interpretations (cluster-bootstrap 95% CI)** | | | | | | | | | |
| ChatGPT — All Interpretations | 180 | 64.4% (52.8–75.6) | 56.7% (41.7–71.2) | 80.0% (61.7–94.0) | 2.83 (1.38–9.56) | 0.54 (0.35–0.80) | 85.0% (68.8–96.6) | 48.0% (27.6–67.3) |  |
| Gemini — All Interpretations | 180 | 53.9% (43.3–63.9) | 52.5% (40.0–64.2) | 56.7% (36.0–76.0) | 1.21 (0.76–2.25) | 0.84 (0.56–1.38) | 70.8% (52.5–87.4) | 37.4% (18.4–56.3) |  |
| All Emergency Specialists | 180 | 75.0% (66.7–83.3) | 67.5% (56.8–77.6) | 90.0% (78.3–98.3) | 6.75 (3.08–32.81) | 0.36 (0.24–0.49) | 93.1% (83.8–99.0) | 58.1% (37.5–75.0) |  |
| All Emergency Residents | 180 | 58.3% (50.0–66.7) | 60.8% (51.7–70.0) | 53.3% (36.9–70.0) | 1.30 (0.92–2.09) | 0.73 (0.50–1.13) | 72.3% (55.8–86.9) | 40.5% (21.1–59.3) |  |
| All Doctors | 360 | 66.7% (61.7–71.9) | 64.2% (58.2–70.0) | 71.7% (62.5–81.7) | 2.26 (1.68–3.51) | 0.50 (0.40–0.61) | 81.9% (68.7–92.1) | 50.0% (30.7–67.0) |  |
| **B. Individual runs / readers (N=36 each; Clopper–Pearson CI, log-method for LR)** | | | | | | | | | |
| ChatGPT — First Interpretation | 36 | 61.1% (43.5–76.9) | 50.0% (29.1–70.9) | 83.3% (51.6–97.9) | 3.00 (0.80–11.31) | 0.60 (0.37–0.96) | 85.7% (57.2–98.2) | 45.5% (24.4–67.8) |  |
| ChatGPT — Second Interpretation | 36 | 61.1% (43.5–76.9) | 54.2% (32.8–74.4) | 75.0% (42.8–94.5) | 2.17 (0.76–6.17) | 0.61 (0.35–1.05) | 81.2% (54.4–96.0) | 45.0% (23.1–68.5) |  |
| ChatGPT — Third Interpretation | 36 | 63.9% (46.2–79.2) | 54.2% (32.8–74.4) | 83.3% (51.6–97.9) | 3.25 (0.87–12.14) | 0.55 (0.33–0.91) | 86.7% (59.5–98.3) | 47.6% (25.7–70.2) |  |
| ChatGPT — Fourth Interpretation | 36 | 66.7% (49.0–81.4) | 58.3% (36.6–77.9) | 83.3% (51.6–97.9) | 3.50 (0.94–12.97) | 0.50 (0.29–0.86) | 87.5% (61.7–98.4) | 50.0% (27.2–72.8) |  |
| ChatGPT — Fifth Interpretation | 36 | 69.4% (51.9–83.7) | 66.7% (44.7–84.4) | 75.0% (42.8–94.5) | 2.67 (0.96–7.40) | 0.44 (0.23–0.85) | 84.2% (60.4–96.6) | 52.9% (27.8–77.0) |  |
| Gemini — First Interpretation | 36 | 58.3% (40.8–74.5) | 62.5% (40.6–81.2) | 50.0% (21.1–78.9) | 1.25 (0.66–2.38) | 0.75 (0.35–1.61) | 71.4% (47.8–88.7) | 40.0% (16.3–67.7) |  |
| Gemini — Second Interpretation | 36 | 41.7% (25.5–59.2) | 29.2% (12.6–51.1) | 66.7% (34.9–90.1) | 0.88 (0.32–2.41) | 1.06 (0.66–1.71) | 63.6% (30.8–89.1) | 32.0% (14.9–53.5) |  |
| Gemini — Third Interpretation | 36 | 52.8% (35.5–69.6) | 58.3% (36.6–77.9) | 41.7% (15.2–72.3) | 1.00 (0.56–1.80) | 1.00 (0.44–2.27) | 66.7% (43.0–85.4) | 33.3% (11.8–61.6) |  |
| Gemini — Fourth Interpretation | 36 | 63.9% (46.2–79.2) | 70.8% (48.9–87.4) | 50.0% (21.1–78.9) | 1.42 (0.76–2.64) | 0.58 (0.25–1.35) | 73.9% (51.6–89.8) | 46.2% (19.2–74.9) |  |
| Gemini — Fifth Interpretation | 36 | 52.8% (35.5–69.6) | 41.7% (22.1–63.4) | 75.0% (42.8–94.5) | 1.67 (0.56–4.95) | 0.78 (0.49–1.24) | 76.9% (46.2–95.0) | 39.1% (19.7–61.5) |  |
| Emergency Specialist-1 | 36 | 77.8% (60.8–89.9) | 70.8% (48.9–87.4) | 91.7% (61.5–99.8) | 8.50 (1.28–56.49) | 0.32 (0.17–0.61) | 94.4% (72.7–99.9) | 61.1% (35.7–82.7) |  |
| Emergency Specialist-2 | 36 | 75.0% (57.8–87.9) | 66.7% (44.7–84.4) | 91.7% (61.5–99.8) | 8.00 (1.20–53.37) | 0.36 (0.20–0.66) | 94.1% (71.3–99.9) | 57.9% (33.5–79.7) |  |
| Emergency Specialist-3 | 36 | 72.2% (54.8–85.8) | 62.5% (40.6–81.2) | 91.7% (61.5–99.8) | 7.50 (1.12–50.24) | 0.41 (0.24–0.70) | 93.8% (69.8–99.8) | 55.0% (31.5–76.9) |  |
| Emergency Specialist-4 | 36 | 75.0% (57.8–87.9) | 70.8% (48.9–87.4) | 83.3% (51.6–97.9) | 4.25 (1.17–15.45) | 0.35 (0.18–0.69) | 89.5% (66.9–98.7) | 58.8% (32.9–81.6) |  |
| Emergency Specialist-5 | 36 | 75.0% (57.8–87.9) | 66.7% (44.7–84.4) | 91.7% (61.5–99.8) | 8.00 (1.20–53.37) | 0.36 (0.20–0.66) | 94.1% (71.3–99.9) | 57.9% (33.5–79.7) |  |
| Emergency Resident-1 | 36 | 63.9% (46.2–79.2) | 62.5% (40.6–81.2) | 66.7% (34.9–90.1) | 1.87 (0.79–4.42) | 0.56 (0.29–1.08) | 78.9% (54.4–93.9) | 47.1% (23.0–72.2) |  |
| Emergency Resident-2 | 36 | 50.0% (32.9–67.1) | 50.0% (29.1–70.9) | 50.0% (21.1–78.9) | 1.00 (0.50–2.00) | 1.00 (0.50–2.00) | 66.7% (41.0–86.7) | 33.3% (13.3–59.0) |  |
| Emergency Resident-3 | 36 | 50.0% (32.9–67.1) | 33.3% (15.6–55.3) | 83.3% (51.6–97.9) | 2.00 (0.50–8.00) | 0.80 (0.55–1.17) | 80.0% (44.4–97.5) | 38.5% (20.2–59.4) |  |
| Emergency Resident-4 | 36 | 63.9% (46.2–79.2) | 83.3% (62.6–95.3) | 25.0% (5.5–57.2) | 1.11 (0.77–1.61) | 0.67 (0.18–2.51) | 69.0% (49.2–84.7) | 42.9% (9.9–81.6) |  |
| Emergency Resident-5 | 36 | 63.9% (46.2–79.2) | 75.0% (53.3–90.2) | 41.7% (15.2–72.3) | 1.29 (0.76–2.19) | 0.60 (0.23–1.57) | 72.0% (50.6–87.9) | 45.5% (16.7–76.6) |  |

Section A: pooled performance over all 180 interpretations per group (360 for All Doctors); point estimates as originally reported, 95% CIs by cluster bootstrap (10,000 resamples, ECG as cluster). Section B: each of the 5 runs (LLMs) / 5 readers (physicians) separately, one decision per ECG (N=36, independent → Clopper–Pearson CIs). PPV, Positive predictive value; NPV, Negative predictive value; CI, Confidence Interval; +LR/–LR = positive/negative likelihood ratio.
