# Supplementary material for: ECG-based detection of occlusion myocardial infarction: a dedicated deep neural network versus multimodal large language models and physicians — a retrospective diagnostic accuracy study
Source: BMC Emerg Med. 2026 Aug 7;26:237. doi: 10.1186/s12873-026-01716-3 (PMC13545760; doi:10.1186/s12873-026-01716-3)
Supplement: Supplementary file 3 [file 12873_2026_1716_MOESM3_ESM.docx]

**Supplementary Table 2.** Pairwise comparisons in the pooled repeated-interpretation analysis

| **Comparison** | **Metric** | **Group 1 (%)** | **Group 2 (%)** | **Difference (95% CI)** | **P (cluster-robust)** |
| --- | --- | --- | --- | --- | --- |
| ChatGPT vs Gemini | **Sensitivity** | 56.7 | 52.5 | +4.2% (-14.3 to 22.6) | 0.658 |
| ChatGPT vs Specialists |  | 56.7 | 67.5 | -10.8% (-30.0 to 8.4) | 0.268 |
| ChatGPT vs Residents |  | 56.7 | 60.8 | -4.2% (-18.0 to 9.7) | 0.556 |
| Gemini vs Specialists |  | 52.5 | 67.5 | -15.0% (-28.5 to -1.5) | **0.030*** |
| Gemini vs Residents |  | 52.5 | 60.8 | -8.3% (-20.1 to 3.4) | 0.165 |
| Specialists vs Residents |  | 67.5 | 60.8 | +6.7% (-9.1 to 22.4) | 0.408 |
| ChatGPT vs Gemini | **Specificity** | 80.0 | 56.7 | +23.3% (4.4 to 42.3) | 0.016* |
| ChatGPT vs Specialists |  | 80.0 | 90.0 | -10.0% (-31.9 to 11.9) | 0.371 |
| ChatGPT vs Residents |  | 80.0 | 53.3 | +26.7% (10.4 to 42.9) | 0.001* |
| Gemini vs Specialists |  | 56.7 | 90.0 | -33.3% (-55.6 to -11.0) | 0.003* |
| Gemini vs Residents |  | 56.7 | 53.3 | +3.3% (-15.0 to 21.7) | 0.722 |
| Specialists vs Residents |  | 90.0 | 53.3 | +36.7% (17.2 to 56.2) | <0.001* |
| ChatGPT vs Gemini | **Accuracy** | 64.4 | 53.9 | +10.6% (-3.6 to 24.7) | 0.144 |
| ChatGPT vs Specialists |  | 64.4 | 75.0 | -10.6% (-25.3 to 4.2) | 0.160 |
| ChatGPT vs Residents |  | 64.4 | 58.3 | +6.1% (-5.6 to 17.8) | 0.306 |
| Gemini vs Specialists |  | 53.9 | 75.0 | -21.1% (-33.1 to -9.1) | <0.001* |
| Gemini vs Residents |  | 53.9 | 58.3 | -4.4% (-14.6 to 5.7) | 0.389 |
| Specialists vs Residents |  | 75.0 | 58.3 | +16.7% (3.5 to 29.9) | 0.013* |

Pairwise comparisons over the pooled interpretations via GEE (identity link, exchangeable correlation) clustered on ECG with cluster-robust SEs; replaces McNemar to account for repeated within-ECG reads and group pairing. * cluster-robust p<0.05. NOTE: Queen of Hearts is not included here — its single-read N=36 data cannot be validly pooled with the 180-read groups; QoH pairwise comparisons are in Table 3 on the matched N=36 footing.
